# Advances in Nondestructive DNA Extraction from Teeth for Human Identification

**DOI:** 10.3390/genes17010113

**Published:** 2026-01-20

**Authors:** Irena Zupanič Pajnič

**Affiliations:** Institute of Forensic Medicine, Faculty of Medicine, University of Ljubljana, Korytkova 2, 1000 Ljubljana, Slovenia; irena.zupanic@mf.uni-lj.si; Tel.: +386-1-543-72-15

**Keywords:** aged teeth, dental cementum, nondestructive DNA extraction, human identification, ethical consideration

## Abstract

This review synthesizes advances in nondestructive DNA extraction from teeth, emphasizing their importance in forensics and archaeogenetics. Because of their mineralized structure and resistance to diagenesis, teeth remain vital for human identification when other tissues are unavailable or degraded. Modern protocols targeting dental cementum have shown high success rates in retrieving nuclear DNA while maintaining specimen integrity, supporting ethical standards, and enabling additional morphological and isotopic analyses. Nondestructive extraction methods produce DNA yields comparable to—or in some archaeological cases, greater than—those of traditional destructive approaches, while ensuring strict contamination control and minimal physical impact. Cementum is a reliable source of DNA in aged and degraded teeth, although the petrous part of the temporal bone still represents the best option under extreme preservation conditions. These results highlight the need for context-specific sampling strategies that balance analytical goals with the preservation of museum collections. Future efforts include testing nondestructive protocols across various forensic scenarios and creating predictive models for DNA preservation. Overall, these developments promote ethical, effective, and sustainable practices in human genomic analysis.

## 1. Introduction

Forensic genetics plays a pivotal role in criminal investigations, disaster victim identification, and humanitarian efforts to locate missing persons, providing decisive evidence when conventional identification methods fail [1,2,3]. In such contexts, skeletal remains often represent the only available biological material, and among skeletal tissues, petrous bones and teeth frequently emerge as the preferred substrate for DNA recovery [4,5]. This preference stems from the unique structural and biochemical properties of teeth: their dense mineral composition, low porosity, and protective enamel layer collectively limit environmental insult and microbial infiltration, thereby preserving DNA over extended periods [6,7].

Teeth comprise several distinct tissues—enamel, dentin, pulp, and cementum—each with differential potential for DNA preservation. Enamel, largely acellular, does not harbor DNA but serves as a protective barrier for the inner tissues [6]. Dentin and pulp, rich in cells, theoretically offer substantial DNA content, particularly in recently deceased individuals. However, their structural characteristics—such as dentin’s tubular architecture and pulp’s high cellularity—predispose them to postmortem degradation, especially in moist or microbially active environments [8]. In aged teeth, cementum (the mineralized outer layer of the tooth root enriched with cementocytes) generally provides higher DNA yields than dentin, making it a preferred target for DNA studies [8,9,10,11,12].

Historically, DNA extraction from teeth and bones has relied on destructive methods, such as complete grinding or drilling to produce powder, followed by demineralization and purification [13,14]. While these methods often yield sufficient DNA for analyses—including short tandem repeat (STR) typing and mitochondrial DNA (mtDNA) sequencing—they irreversibly destroy the specimen, precluding subsequent morphological, isotopic, or radiocarbon investigations. Such destruction raises ethical concerns, particularly for culturally sensitive remains, rare archaeological specimens, or remains intended for repatriation [15,16]. In response, the field has increasingly embraced minimally invasive and nondestructive DNA extraction strategies. Over the past decade, dental cementum has emerged as a resilient reservoir of nuclear DNA, even in aged, degraded, or archaeologically recovered teeth [10,11,17]. Advances in extraction protocols—including demineralization, enzyme-assisted lysis, stringent contamination control, and automation—have achieved high DNA recovery rates while preserving tooth morphology and minimizing specimen damage [12,18].

Minimally destructive cementum extraction showed high efficiency. When 30 ancient teeth and nine corresponding petrous bones were analyzed, DNA yields from targeted cementum were comparable to those from conventional destructive methods, preserving the tooth for subsequent morphological or biochemical analyses [18]. Broader osteological studies, such as the analysis of 62 archaeological canines from the 13th to 19th centuries, reported informative STR profiles in 74% of cases using nondestructive cementum-focused methods, with nearly half producing complete autosomal profiles [12]. Subsequent destructive grinding of residual tissue left after cementum DNA extraction from these same teeth yielded only 8% informative profiles, confirming cementum as the principal nuclear DNA reservoir in aged teeth. In curated collections, where conservation is paramount, nondestructive cementum extraction should replace traditional grinding, cutting, or drilling of teeth [12].

Given these developments, forensic laboratories and archaeogenetic programs must adopt context-sensitive sampling strategies, selecting the optimal tissue (cementum versus petrous bone) and extraction method (nondestructive versus destructive) according to preservation state, constraints in maintaining sample integrity, and planned downstream analyses. This review compiles methodological advances, tissue-specific preservation data, and practical implementation considerations, providing a framework for ethical, effective, and sustainable DNA recovery from human teeth.

## 2. Role of Teeth in Forensic Identification

**Figure 1:**
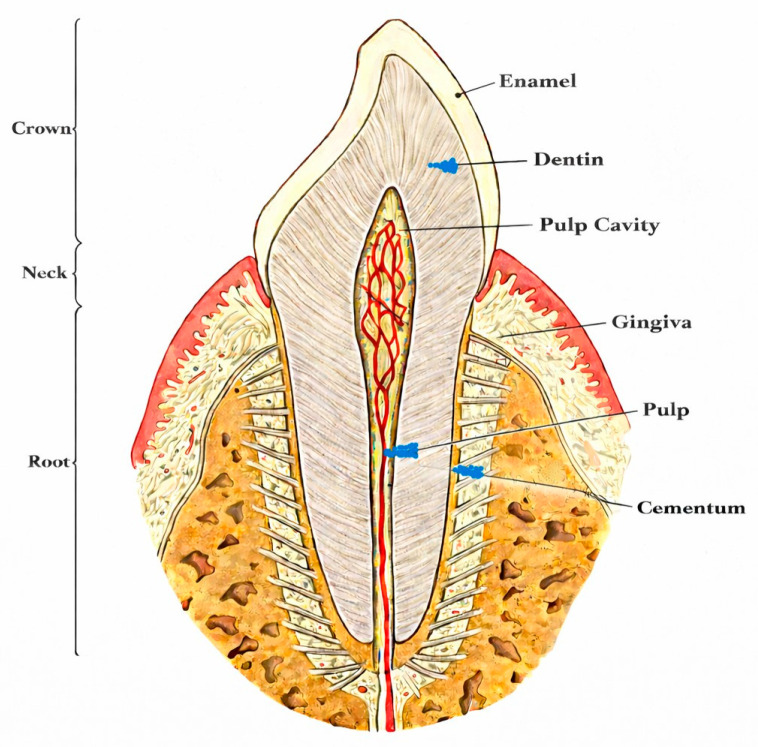
Tooth anatomy.

Teeth are among the most durable elements in human skeletal remains, frequently surviving in contexts where bones are fragmented or entirely lost. This durability is attributed to their composition (see Figure 1) and microarchitecture: enamel comprises approximately 96% mineral content, while dentin and cementum contain around 70% mineral, creating a dense, low-porosity structure that limits penetration by environmental inhibitors and microorganisms [6,17]. Consequently, teeth can outperform many skeletal elements in DNA preservation under specific taphonomic conditions. Nonetheless, other dense bone elements, particularly the petrous portion of the temporal bone, often yield higher DNA content, especially in poorly preserved remains [19,20]. Sampling petrous bone, however, is highly invasive and frequently constrained by ethical considerations [10,21,22,23].

Within the tooth, different tissues present unique opportunities and limitations for DNA extraction. The pulp, rich in nucleated cells, is typically the initial target in modern teeth due to its high DNA potential. However, its susceptibility to postmortem degradation—through putrefaction, microbial infiltration, and enzymatic decomposition—renders DNA highly labile, particularly in moist or warm burial environments [6,24]. Dentin, containing long odontoblastic processes within a tubular network, provides structural protection but remains permeable, allowing infiltration of fluids, microbes, and inhibitory compounds, vulnerabilities that increase over extended postmortem intervals [8].

Cementum, by contrast, presents a highly resilient alternative. As a moderately mineralized tissue enveloping the tooth root, cementum contains cementocytes embedded in lacunae, forming a dense matrix that restricts exposure to environmental insult. This structural stability, coupled with its lower porosity compared with dentin, mitigates DNA degradation and microbial infiltration, often preserving nuclear DNA even in ancient or degraded teeth [10,17,25].

In forensic or archaeological scenarios, where remains may be repatriated, curated, or subjected to multiple analyses, the ability to extract DNA without compromising the specimen is ethically and practically invaluable. Cementum-targeted, minimally destructive methods provide an optimal balance between DNA recovery and preservation of tooth integrity [15,16].

While the petrous bone remains the gold standard for maximal DNA yield in poorly preserved remains [19,20], teeth—and specifically cementum—offer a pragmatic, noninvasive, and often highly effective alternative in many forensic and archaeogenetic contexts. Substrate selection should therefore follow a context-driven strategy, accounting for preservation state, constraints in maintaining sample integrity, and the requirement for additional analyses.

## 3. DNA Preservation Across Dental Tissues

DNA preservation within teeth is highly heterogeneous and depends on tissue type, postmortem interval, burial environment, individual’s age, tooth type, and dental pathology. In recently extracted teeth, such as those obtained for clinical purposes, pulp and dentin may yield high-quality nuclear DNA due to their high cellular content and structural integrity. However, in aged or archaeological specimens, these tissues frequently fail to preserve amplifiable DNA, whereas cementum demonstrates remarkable resilience [8,10,11,12,17].

Mechanistically, cementum’s superior DNA preservation can be attributed to its cellular architecture. Cementocytes reside within lacunae embedded in a dense mineral matrix, protecting from enzymatic degradation, hydrolysis, and microbial infiltration. Its moderate yet stable mineralization further shields nucleic acids bound to hydroxyapatite from chemical and physical decay. In contrast, dentin’s tubular structure permits fluid flow and may harbor microbial communities that accelerate degradation, while pulp is highly susceptible to early postmortem decay due to microbial activity and absence of hard matrix protection [6,9,26].

Age-related changes also influence DNA distribution within teeth. The cementum layer thickens over time due to continuous deposition, increasing the volume of cellular cementum. This structural adaptation provides an additional source of mitochondrial and nuclear DNA even as pulp volume and nuclear DNA content decline. Consequently, older teeth, despite reduced pulp-derived DNA, can still serve as important reservoirs for forensic and archaeogenetic studies. Empirical evidence supports cementum as a more reliable source than dentin or pulp, particularly in ancient or highly degraded specimens [8,9,10,11,25]. This age-dependent variation underscores the necessity of considering tooth type and age when selecting samples for DNA extraction.

Environmental factors exert a significant influence on DNA preservation. Variables such as soil pH, moisture, microbial load, temperature fluctuations, burial depth, and postmortem interval collectively determine degradation rates. Acidic or highly variable environments accelerate hydrolysis and depurination, reducing DNA integrity. Elevated moisture and microbial activity further contribute to degradation through enzymatic and oxidative mechanisms [22,27,28,29]. Dental pathology, including caries or root resorption, may compromise cementum integrity and thereby reduce DNA preservation.

Developmental and ontogenetic factors further complicate DNA recovery. Recent studies comparing permanent and deciduous teeth in both adults and nonadults demonstrate higher DNA yields in permanent adult teeth, likely due to thicker cementum layers and fully developed roots [30]. These findings have direct implications for forensic and archaeogenetic investigations, particularly when juvenile remains are involved [20,31].

Collectively, these data emphasize that cementum, owing to its structural robustness and biochemical stability, represents the most reliable substrate for nuclear DNA preservation in aged teeth. Context-sensitive sampling strategies, accounting for environmental and developmental factors, are essential to optimize DNA recovery (see Table 1).

**Table 1:** Summary of nuclear DNA preservation in dental tissues in aged samples.

Tissue	Nuclear DNA Preservation	Typical STR Success	Key Modifiers	Notes
Cementum	High; frequent recovery of nuclear DNA	High	Cementum thickness (apical), microenvironment, demineralization efficiency	Cementocytes as DNA source; robust to diagenesis
Dentin	Moderate–Low in aged samples; better in modern teeth	Variable; reduced in archaeological contexts	Tubule permeability; pathology; age	DNA bound to mineral/collagen; sensitive to diagenesis
Pulp	Low in aged samples; high in fresh teeth	Poor in archaeological samples	Moisture, microbial activity, and ante mortem procedures	Rapid putrefaction; occasionally preserved under desiccation

## 4. DNA Extraction from Teeth

Established DNA extraction techniques from teeth have traditionally been destructive, involving highly invasive procedures such as complete grinding or pulverization of the tooth [13,14,32]. Less invasive approaches include scraping the cementum layer with a sterile scalpel [33,34], targeted access to dental pulp through precise drilling [34,35], or partial drilling of the tooth root [36]. Several nondestructive protocols have been developed for DNA recovery from ancient teeth [15,18,37,38,39]. Recent advances incorporate automation for DNA purification using commercially available forensic extraction kits, standardizing workflows while minimizing contamination risks [12].

**Destructive extraction methods** generally involve grinding the entire tooth into powder, followed by decalcification using EDTA to release DNA embedded in the mineral matrix. Subsequent lysis buffers containing detergents such as SDS and proteinase K degrade cellular material, and purification is typically achieved through phenol–chloroform extraction or silica-based spin columns [40,41]. Rohland and Hofreiter [13] developed a protocol optimized for highly degraded archaeological material, maximizing recovery of amplifiable ancient DNA (aDNA) while minimizing PCR inhibitors. This involves mechanical cleaning of the tooth surface, grinding under sterile conditions, decalcification in EDTA, enzymatic digestion with proteinase K, and DNA purification via silica columns using chaotropic agents such as guanidinium thiocyanate. This approach is optimized for short, damaged DNA molecules and has been widely applied in paleogenetic studies targeting both mitochondrial and nuclear DNA [13].

Dabney et al. [42] introduced an optimized silica-based method for extracting highly fragmented DNA, enabling recovery of molecules as short as 30 bp. The protocol increases binding buffer-to-sample ratios, employs spin columns with extended reservoirs, and enhances the retention of ultrashort DNA fragments. After DNA binding, washing with ethanol removes residual inhibitors, and elution in low-salt buffer provides DNA suitable for downstream analyses [42].

Zupanič Pajnič [14] demonstrated that automation can further improve DNA recovery. Following chemical cleaning and UV irradiation to reduce contamination, teeth are ground under liquid nitrogen using a bead mill to prevent thermal damage. Decalcification with EDTA, lysis with proteinase K and DTT, and automated purification using magnetic silica-coated particles allow high-quality DNA recovery from minimal material, even in Second World War [43] and archaeological samples [44]. This method minimizes manual handling and avoids hazardous solvents [14].

**Partially destructive methods** aim to reduce physical damage while accessing DNA-rich tissues. These include pulp drilling or scraping of the cementum layer [25,33,34,35]. While less invasive than full grinding, they still modify the tooth structure and may fail to access the DNA-richest tissue if sampling is not accurately targeted [8,11]. Operator skill and technique variability further influence DNA yield.

Wei et al. [34] compared two minimally destructive approaches. The first employed orthograde occlusal access to the pulp chamber using diamond burs with water cooling to prevent thermal DNA damage. Biological material was collected with barbed broaches and sequential endodontic files, and the cavity was restored with composite resin. While technically demanding and time-consuming (30–60 min per tooth), this method can yield high DNA quantities in well-preserved teeth. The second approach involved gentle scraping of root cementum with a sterile surgical blade, yielding 5–10 mg of material per tooth within 10 min. Cementum sampling demonstrated superior DNA quality and STR profile completeness, especially in degraded specimens, while preserving tooth morphology. The authors recommend prioritizing cementum scraping for forensic and archaeological contexts where morphological integrity is critical, reserving pulp sampling for supplementary analyses such as pathogen detection [34].

Inostroza et al. [35] described controlled pulp and cementum sampling under sterile conditions. Teeth were hydrated externally, and pulp was accessed via perforation of the occlusal surface and apical root using diamond burs guided by radiographic measurements. Pulp extraction employed an automated endodontic file, while cementum slices were carefully scraped from the radicular surface. Both tissue types were collected in sterile containers and rinsed with DNA-free water before extraction. This workflow allows simultaneous collection of multiple tissue types while preserving overall tooth morphology for forensic or anthropological applications [35].

Higgins et al. [25] manually sampled cementum by scraping the outer root surface with a sterile disposable scalpel, lysing 15–50 mg of tissue overnight in ATL buffer with Proteinase K without decalcification. Correa et al. [33] applied a powder-free, minimally destructive approach, covering the crown with parafilm, demineralizing root regions in EDTA, scraping 20 mg of cementum, and purifying DNA using the EZ1 DNA Investigator Kit.

**Nondestructive extraction methods** represent a paradigm shift. Rohland et al. [37] developed a protocol for museum specimens that allows DNA recovery without altering tooth morphology, involving immersion in a guanidinium thiocyanate-based buffer and gentle rotation at 40 °C for up to seven days. DNA is subsequently purified using silica-based methods, achieving amplifiable mtDNA in 88% of specimens. Bolnick et al. [38] extended nondestructive approaches to human skeletal remains, obtaining both mitochondrial and nuclear DNA while preserving structural integrity. Dobosz et al. [39] implemented EDTA-based buffers, proteinase K, and controlled elution via syringe systems, achieving success rates slightly below destructive methods but maintaining morphological integrity.

Harney et al. [18] described a cementum-focused, minimally destructive protocol involving surface decontamination, parafilm protection of crowns, selective root demineralization with EDTA and proteinase K, and standard aDNA purification. DNA yields were comparable to destructive methods, with preserved morphology suitable for subsequent isotopic or morphological analyses.

Large-scale evaluations, including 62 archaeological canine teeth, confirmed high STR success using nondestructive cementum-focused methods, with nearly half producing complete autosomal profiles [12]. Subsequent destructive grinding of the same teeth yielded informative profiles in only a small fraction of cases, reinforcing cementum as the principal nuclear DNA source in aged teeth.

Nondestructive methods are compatible with forensic-grade automation (silica columns or magnetic bead-based systems), optimized elution volumes, and trace DNA workflows. Teeth remain intact or minimally altered, enabling downstream analyses. Limitations include minor surface damage in friable specimens and restricted access to noncementum tissues, which may contain pathogen DNA or other targets.

Recent research demonstrates that cementum-based extraction often yields higher-quality DNA than pulp or dentin, particularly in environmentally stressed teeth [34]. Tissue-specific preservation dynamics, refined extraction protocols, contamination control, and automation collectively support the adoption of nondestructive cementum extraction as a first-line strategy in forensic and archaeogenetic workflows.

## 5. Comparative Evaluation of Extraction Approaches

The choice of extraction method significantly influences DNA yield, quality, and downstream genotyping success. Fully destructive, partially destructive, and nondestructive approaches were developed for forensic and archaeological applications [12,13,34,35].

**Destructive grinding methods** provide high DNA yields due to complete access to all mineralized and soft tissue compartments. Dabney et al. [42] demonstrated that whole-tooth powdering recovers ultrashort DNA fragments, critical for highly degraded samples. Loreille et al. [40] and Amory et al. [41] confirmed that grinding followed by EDTA decalcification and silica column purification achieves reproducible results across archaeological and forensic specimens. However, the major limitation is the irreversible loss of morphological integrity, preventing subsequent osteological, isotopic, or museum-based analyses. In addition, full grinding introduces increased risk of laboratory contamination if strict clean-room protocols are not followed.

**Partially destructive methods**, such as pulp chamber drilling or cementum scraping, balance DNA recovery and preservation of tooth structure. Wei et al. [34] showed that controlled pulp access can provide comparable DNA yields to full grinding in well-preserved teeth but requires advanced technical skills and is time-intensive. Cementum scraping, by contrast, is rapid, reproducible, and yields high-quality DNA from minimal material. Several comparative studies [11,25,33] demonstrated that cementum-derived DNA consistently produces more complete STR profiles than dentin or pulp in aged or environmentally compromised teeth. Nonetheless, these methods still involve some alteration of the tooth root, which may be undesirable in certain forensic or museum contexts.

**Nondestructive extraction protocols** offer the advantage of preserving tooth morphology while still recovering nuclear and mtDNA suitable for forensic and aDNA analyses. Rohland et al. [37] first established this approach, demonstrating successful DNA recovery from museum specimens without visible morphological change. Subsequent refinements, including selective cementum demineralization and automated purification [18,39], have improved yield and reproducibility. Zupanič Pajnič and Leskovar [12] evaluated archaeological teeth using cementum-focused nondestructive extraction, achieving high STR typing success. Comparative testing revealed that nondestructive cementum methods often outperform destructive grinding in producing intact STR profiles from aged samples, likely due to preferential DNA preservation in the cementum layer [45].

A systematic review and meta-analysis examined forensic DNA extraction methods for hard tissues, with particular attention to teeth as a source of genetic material. Across studies, targeted sampling of cementum or pulp consistently improved DNA yield and STR profiling success compared to pulverizing whole teeth, which often introduces variability and contamination [46].

DNA quality assessment across methods involves quantification of total nuclear and mtDNA, assessment of fragment length, inhibitor presence, and success in STR or single-nucleotide polymorphism (SNP) amplification. Dabney et al. [42] highlighted that ultrashort fragments (<50 bp) are better retained in silica-based extraction from powdered teeth, whereas partially destructive cementum scraping preferentially captures slightly longer, higher-quality DNA suitable for routine forensic STR analysis. Harney et al. [18] demonstrated that nondestructive cementum extraction maintains fragment integrity while minimizing inhibitory compounds, facilitating efficient PCR amplification.

Contamination considerations also differ between methods. Destructive grinding exposes all internal tooth surfaces, increasing the likelihood of exogenous DNA incorporation if stringent decontamination protocols are not followed. Partially destructive and nondestructive approaches limit exposure, particularly when crown surfaces are protected with parafilm or UV-irradiated before extraction [18,33]. Automated purification workflows further reduce manual handling and the risk of laboratory contamination, enhancing reliability for forensic casework and archaeogenetic research [12].

Time and resource efficiency are other factors. Fully destructive methods require extended preparation, grinding, and decalcification periods, while partially destructive approaches, especially cementum scraping, can be completed rapidly (5–10 min per tooth) with minimal equipment [34]. Nondestructive cementum-focused protocols may require longer incubation (several hours to a few days) for adequate DNA release but offer a trade-off in preserving tooth morphology. The choice of approach thus depends on sample context, preservation status, downstream analytical requirements, and time constraints.

Integrating the extraction strategy with automated workflows, contamination control, and DNA quantification optimizes overall success rates. Cementum-focused nondestructive methods are increasingly recognized as a reliable, reproducible, and minimally invasive standard for forensic and archaeogenetic applications, providing high-quality DNA while maintaining sample integrity for subsequent morphological or isotopic analyses (see Table 2) [47].

**Table 2:** Summary of destructive, partially destructive, and nondestructive approaches of DNA extraction from teeth.

Approach	DNA Yield	Specimen Impact	Contamination Risk	Limitations
Destructive	High	Complete loss	High	Loss of morphology, ethical concerns
Pulp Drilling (Partial)	Moderate	Visible openings	Moderate	Pulp degradation, variable success
Cementum Scraping (Partial)	Moderate-High	Minor surface loss	Moderate	Operator-dependent, aerosol generation
Nondestructive	High	Minimal	Low	Friable teeth damage, pathogen DNA reduced

## 6. Advantages of Nondestructive Cementum Extraction

Nondestructive cementum extraction provides multiple advantages spanning scientific, operational, and ethical domains, establishing it as a preferred method for forensic and archaeogenetic investigations.

**The primary advantage is the preservation of anatomical integrity.**

By avoiding complete tooth destruction, nondestructive methods enable subsequent analyses such as morphological assessment, isotopic studies, and radiocarbon dating, while also supporting repatriation of remains to families or curation in museum collections [15,16,48]. This is particularly relevant for culturally sensitive specimens, rare archaeological finds, and cases involving repatriation where irreversible damage is ethically unacceptable.

**Another key advantage is the maximized recovery of nuclear DNA.**

Dental cementum has been consistently demonstrated to retain higher-quality nuclear DNA than pulp or dentin in aged, degraded, or archaeologically recovered teeth [10,12,17]. Targeting the cementum ensures optimal recovery even when environmental or taphonomic conditions are suboptimal, including prolonged burial, microbial activity, or chemical exposure. The dense mineralization and lacunar embedding of cementocytes confer remarkable protection against hydrolysis, enzymatic degradation, and microbial infiltration, thereby preserving DNA integrity over extended postmortem intervals.

**Reduced contamination risk and operational efficiency** further enhance the appeal of nondestructive approaches. By minimizing manual handling and utilizing automated purification systems, such as silica-based columns or magnetic bead platforms, these protocols decrease the likelihood of modern DNA contamination, standardize workflow reproducibility, and support high-throughput processing [12,18]. Integration of elimination databases (reference DNA profiles of laboratory staff and other handlers to identify and exclude contamination) and traceability protocols ensures rigorous quality control, which is essential for forensic casework and culturally sensitive samples.

**Compatibility with multi-omic and downstream analyses** represents an additional advantage. Teeth processed using nondestructive methods remain suitable for complementary isotopic, radiocarbon, or protein-based analyses, enabling a comprehensive reconstruction of individual life histories while conserving limited biological material. Such an approach aligns with contemporary standards in bioarchaeology, forensic genetics, and museum curation, maximizing the scientific value obtained from a single specimen [16].

**Ethical considerations** are embedded in the practice of nondestructive cementum extraction. The method minimizes the conflict between obtaining high-quality genetic data and preserving culturally or historically significant human remains. By reserving destructive sampling for cases where nondestructive methods fail or where maximal genome-wide data are necessary, laboratories can adhere to ethical guidelines and maintain stakeholder trust. This balance is particularly critical when working with rare, fragile, or repatriation-bound specimens.

**Operational versatility and scalability** further reinforce the method’s utility. Nondestructive cementum extraction protocols can be implemented across diverse laboratory settings, from small-scale forensic laboratories to large archaeogenetic research projects. The approach is compatible with both manual and automated systems, and can accommodate varying sample throughput, ensuring efficiency without compromising DNA quality or structural preservation.

Nondestructive cementum-focused extraction integrates scientific rigor, operational efficiency, and ethical responsibility. It allows for reliable nuclear DNA recovery while preserving anatomical integrity, facilitating subsequent analyses, reducing contamination risk, and ensuring ethical compliance. These advantages collectively position nondestructive cementum extraction as a first-choice method in forensic, archaeological, and museum-based workflows, supporting a tiered sampling strategy that prioritizes conservation without sacrificing analytical quality.

## 7. Comparative Analysis of Petrous Bones and Tooth Cementum

Teeth, and in particular dental cementum, provide a minimally destructive yet highly informative source of DNA; however, the petrous portion of the temporal bone remains the gold standard for aDNA recovery, particularly in poorly preserved specimens [10,19,20,21,22,23,31,44]. The comparative evaluation of these substrates is essential for designing context-sensitive sampling strategies in both forensic and archaeogenetic research.

Hansen et al. [10] conducted a comprehensive study of 34 skeletons spanning diverse temporal and geographic contexts, including the Bronze Age of Central Asia, the Viking Age in England, later historical periods in Denmark, and cremated remains from the Danish Iron Age. Their analysis highlighted that, under conditions of good dental preservation, teeth performed as effectively as—or occasionally better than—petrous bones in yielding DNA. Conversely, in cases of poor dental preservation, petrous bones consistently outperformed tooth cementum, underscoring the influence of preservation state on substrate selection. Visual inspection of teeth, particularly evaluation of cementum integrity and root texture, proved a reliable predictor of DNA quality: well-preserved teeth with intact, compact cementum layers yielded high DNA, whereas brittle, chalky roots with eroded cementum performed poorly. This finding is significant because petrous bone quality cannot be assessed without invasive sampling, whereas tooth condition is readily observable, allowing preliminary evaluation before destructive intervention [10]. Further analysis revealed differences in DNA degradation patterns between substrates. Petrous bones exhibited higher levels of cytosine-to-thymine deamination, indicative of chemical decay, despite retaining higher absolute DNA yields. Fragment length distribution analyses suggested similar mean fragment sizes between teeth and petrous bone, although teeth showed a more pronounced 10 base-pair periodicity associated with nucleosome protection, reflecting differential chromatin preservation. Additionally, teeth displayed higher mitochondrial-to-nuclear DNA ratios, particularly in poorly preserved specimens, emphasizing the resilience of circular mitochondrial genomes relative to nuclear DNA. From a practical standpoint, this characteristic facilitates mitochondrial haplogroup determination even in contexts where nuclear genome recovery is limited, whereas petrous bone provides a more balanced nuclear and mitochondrial representation suitable for genome-wide analyses [10].

A subsequent comparative study of 60 archaeological skeletons from two sites dating from the 13th to 19th centuries reinforced these observations [49]. Petrous bones generally provided higher DNA yields and greater amplification success than tooth cementum, although this advantage was largely confined to the subset of specimens with severely degraded dental tissue. Excluding poorly preserved teeth (15 teeth) from the analysis revealed no significant difference in STR typing success between the two substrates, demonstrating that well-preserved teeth can achieve performance comparable to petrous bones while avoiding the destructive impact associated with cranial sampling. This study also highlighted differences in DNA degradation indices, with petrous bones exhibiting more extensive fragmentation—likely a consequence of mechanical stresses during grinding—whereas nondestructive cementum methods preserved structural integrity and minimized additional DNA damage [49].

The operational advantages of teeth are noteworthy. Nondestructive extraction from dental cementum shortens processing time, reduces contamination risk, and obviates the need for specialized equipment and liquid nitrogen. Morphological features critical for anthropological and isotopic studies remain intact, enhancing the utility of specimens for multi-disciplinary research. Teeth are also inherently less susceptible to exogenous contamination due to their dense enamel layer, which acts as a barrier to environmental DNA. These features collectively position tooth cementum as an ethically and scientifically sound alternative to petrous bone in contexts where preservation is adequate [49].

Nevertheless, the superiority of the petrous bone under conditions of extreme degradation, including cremated or heavily compromised specimens, remains indisputable. Petrous bone consistently demonstrates higher DNA yield, greater fragment length, and increased complexity under harsh taphonomic conditions [10,18,20,31]. Consequently, a context-driven, tiered sampling strategy is recommended: prioritize nondestructive cementum extraction when teeth are well preserved, but resort to petrous bone sampling when maximal DNA recovery is critical or when dental preservation is poor.

In conclusion, both tooth cementum and petrous bone are validated as exceptional DNA reservoirs, each with distinct advantages and limitations. Cementum-focused nondestructive extraction maximizes ethical compliance and preserves specimen integrity while delivering high-quality DNA in many contexts. Petrous bone remains the preferred substrate when extreme preservation challenges demand maximal yield [10,18,20,31]. Adopting a tiered, context-sensitive approach ensures that sampling decisions balance scientific objectives with conservation ethics, particularly when working with rare or culturally significant human remains.

## 8. Discussion

The emergence of cementum-focused, nondestructive DNA extraction represents a pivotal advancement in forensic genetics and archaeogenetics, effectively reconciling the dual imperatives of scientific rigor and ethical stewardship. Evidence from multiple studies consistently demonstrates that cementum, particularly the apical cellular layer, functions as a stable repository of nuclear DNA in aged and degraded teeth [10,12,17,18,25].

Mechanistically, the resilience of cementum is readily explained by its microarchitecture. Cementocytes are embedded within lacunae surrounded by a dense mineral matrix, shielding DNA from enzymatic hydrolysis, microbial infiltration, and other degradative processes. The moderate yet stable mineralization constrains fluid diffusion, further protecting nucleic acids bound to hydroxyapatite. By contrast, pulp lacks a protective mineral matrix, and dentin’s tubular structure permits fluid and microbial penetration, rendering these tissues more susceptible to post-mortem degradation [6,9].

Methodological innovations have significantly enhanced the efficacy and accessibility of nondestructive extraction. The protocol described by Harney et al. [18] demonstrates that DNA yield and quality from cementum can match those of traditional destructive approaches, while preserving tooth morphology and enabling subsequent morphological, isotopic, or biochemical analyses, and radiocarbon dating. Complementing these findings, recent empirical data from 62 archaeological teeth revealed a high STR typing success rate, underscoring the practical reliability of cementum-based extraction [12].

Comparative analyses of DNA from cementum versus pulp after short post-mortem intervals (≤3 months) indicate that although pulp may occasionally yield higher quantities of DNA, cementum consistently provides superior DNA integrity, more complete STR profiles, and the additional benefit of preserving tooth structure [34]. These technical advantages are paralleled by ethical and practical considerations. Preservation of anatomical integrity facilitates repatriation, museum curation, and multi-disciplinary downstream analyses, thereby maximizing scientific return from limited or sensitive specimens [15,16]. Reduced manual handling combined with automated workflows minimizes contamination risk, supporting high-throughput applications suitable for both forensic casework and archaeogenetic investigations [12,18]. The use of elimination databases remains critical for traceability and quality assurance [22,50,51].

Despite these advances, several limitations and challenges persist. DNA preservation is not uniform across teeth; cementum thickness, root morphology, dental pathology, and taphonomic factors influence DNA yield and quality. For example, permanent teeth generally exhibit thicker cementum and more fully developed roots than deciduous teeth, yielding superior DNA recovery [30]. Furthermore, environmental and burial conditions—including soil pH, microbial load, moisture, and temperature—modulate DNA stability. In acidic, wet, or microbially active contexts, even cementum may undergo significant degradation, necessitating alternative or supplementary sampling from petrous bone or other dense skeletal elements [19,22,27,28].

Additional considerations include studies of pathogen DNA or other molecular targets that require access to pulp or dentin. In such cases, cementum-only sampling may be insufficient, and more invasive techniques or combined strategies may be required [18,42,50,52]. The advent of next-generation sequencing (NGS), SNP arrays, and whole-genome sequencing further underscores the need for adequate fragment length, authenticity, and molecular complexity [44]. Cementum-based extraction has demonstrated compatibility with these requirements [18], but further validation across diverse environmental and forensic scenarios—including burned, immersed, or chemically treated remains—is warranted.

Future directions include the development of predictive models for DNA preservation that integrate intrinsic factors—such as tooth type, cementum thickness, age, and dental pathology—with extrinsic environmental metadata, including soil chemistry, burial depth, moisture, and temperature [29]. Such models would optimize sampling strategies, reduce failed extractions, and enhance resource efficiency. Multi-omics frameworks, which combine nuclear DNA, mtDNA, proteins, and isotopes from a single tooth, represent a promising avenue for maximizing scientific output while minimizing destruction.

Finally, comparative research on petrous bones versus tooth cementum in archaeological skeletons confirms that nondestructive cementum extraction should be considered a first-choice method when preservation is adequate and structural integrity matters [49]. Only when cementum yields are insufficient or when comprehensive genome-wide data are required should invasive sampling of petrous bone be employed.

Cementum-focused nondestructive DNA extraction represents a significant advancement in forensic and archaeogenetic practice. It preserves specimens, reduces contamination risk, enables multi-omic analyses, and yields high-quality DNA. Ongoing refinement, contextual validation, and integration with modern sequencing technologies and predictive modeling will further establish cementum-based approaches in human identification and aDNA research.

## 9. Conclusions

Cementum-focused, nondestructive DNA extraction represents a critical innovation in forensic and archaeogenetic sciences, reconciling the imperatives of scientific rigor, ethical stewardship, and specimen preservation [6,48]. Across diverse temporal, environmental, and taphonomic contexts, cementum consistently demonstrates high nuclear DNA stability, offering reliable recovery of STR profiles, SNP data, and mitochondrial sequences while maintaining tooth morphology [8,11,25]. The protective embedding of cementocytes within the mineralized matrix explains both the integrity and longevity of DNA, rendering cementum a superior substrate compared with pulp or dentin in degraded or aged teeth [9,15].

Empirical evidence from modern forensic casework and archaeological assemblages demonstrates that cementum-based approaches achieve DNA yields and quality comparable to or exceeding traditional destructive methods, with additional advantages of reduced contamination risk, compatibility with high-throughput workflows, and retention of morphological and isotopic information [18,35,38]. These benefits are particularly salient in contexts where specimen preservation is paramount, including museum collections, repatriation cases, or sensitive archaeological sites [15,48].

The nondestructive approach also aligns with ethical standards and professional guidelines, ensuring that scientific inquiry does not compromise the structural integrity of culturally or historically significant specimens [15,16]. By enabling multi-omic analyses from a single tooth, including DNA, protein, and isotopic profiling, cementum-focused extraction maximizes data yield while minimizing material consumption [30,49].

Nevertheless, several constraints must be acknowledged. DNA preservation in cementum is influenced by tooth type, cementum thickness, root morphology, age at death, dental pathology, and, most importantly, environmental conditions such as soil pH, microbial activity, and burial moisture [10,36,42]. In cases of extreme degradation or specialized analytical needs—such as pathogen recovery or comprehensive genome-wide sequencing—cementum extraction may be complemented by petrous bone sampling or other skeletal sources [13,49]. Ongoing refinement of extraction protocols, coupled with predictive modeling that integrates intrinsic and extrinsic preservation factors, will optimize sampling strategies and improve success rates [12,34].

In conclusion, cementum-focused, nondestructive DNA extraction establishes a paradigm of methodological excellence that harmonizes ethical, and scientific priorities [15,16]. Its application in forensic investigations, archaeogenetics, and bioarchaeology facilitates reliable human identification, population genetic studies, and multi-disciplinary research, setting a standard for future investigations where preservation, data quality, and analytical versatility are equally critical [11,25,49]. Continued validation across diverse environmental contexts and integration with NGS platforms will consolidate its role as a first-choice strategy for ancient and contemporary dental DNA recovery [18,32].

## Data Availability

No new data were created or analyzed in this study. Data sharing is not applicable to this article.
